# The Relation between the Radial Collapse and the Number of Metaphyseal Screws for Distal Radius Fractures

**DOI:** 10.5704/MOJ.2211.006

**Published:** 2022-11

**Authors:** A Askin, C Aldemir, F Duygun, V Nabi

**Affiliations:** Department of Orthopaedics and Traumatology, Antalya Training and Research Hospital, Antalya, Turkiye

**Keywords:** distal radius fracture, locking screw, volar plate, radiological outcome, loss of reduction

## Abstract

**Introduction:**

The purpose of this study is the evaluation of radial collapse, based on the number of screws used in the metaphyseal region and by distal dorsal distance (DDD) and lunate facet distance (LFD) measurement.

**Materials and methods:**

Between 2015 and 2019, 60 patients who were being treated with volar locking plates due to isolated distal radius fracture were evaluated. Control radiographs were taken on the first day and at 3rd-, 6th- and 12th-month follow-ups. Distal dorsal cortex distance and lunate facet distance were measured in the lateral radiographs. The number of screws used in the metaphyseal region was also evaluated. According to the number of screws, the amount of collapse was analysed based on both the LFD and the DDD.

**Results:**

The mean age of patients was 43.5±12.7 years. Thirty-three of the patients included in the study were male and 27 were female, and the minimum follow-up period was one year. According to the mean number of screws, groups were defined as up to 6 screws or 7 screws and above. There was a statistically significant difference between the groups in terms of DDD collapse at the 6th-month and 12th-month follow-ups (p<0.005). It was observed that the radial collapse and decrease in DDD and LFD were lower in plates with seven screws and above.

**Conclusion:**

Decreases in either DDD or LFD, or radial collapse were observed less in patients who had seven or more metaphyseal screws inserted. These findings might be useful for surgeons treating distal radius fractures to reduce radial collapse.

## Introduction

Distal radius fractures account for 0.17% of all skeletal fractures and 75% of forearm fractures and continue to be challenging for orthopaedic surgeons. The incidences are 9/ 10,000 in males and 8/ 10,000 in females^1,2^. Approximately half of the distal radius fractures are stable fractures. Despite the initial successful closed reduction of displaced fractures, it should be taken into consideration that 40% of such fractures lose reduction and may require surgical intervention^3^. There are many established causes of radial collapse after volar plate fixation of distal radius fractures. These reasons have been categorised into two groups.

First, locking screw bridging the fracture gap; plate-bone distance of more than 5mm; plate material, dimension and shape; screw configuration; plate length and working length; mis-insertion; and angulated insertion of the screwhead can cause the implant-related radial collapse and failure^4-11^. In addition, fracture configuration and screw-bone interface can pose bone-related failure^5,12,13^. Because of its ability to preserve fracture stability, the management of distal radius fractures using volar plate fixation has become a commonly preferred treatment option^14-16^.

Once the evolution of plates and screws provides an opportunity for anatomical reduction in the distal radius, maintaining the reduction presents the next challenge to the surgeon as implant-related complications tend to increase^2,17^. Previous researchers have looked into various factors that should be considered to maximise stable internal fixation when using locked volar plates in distal radius fractures.

The surgical anatomy of the volar radial surface should be taken into consideration to achieve satisfactory outcomes. The volar locking plate should be implemented based on five crucial anatomic landmarks: the volar radial tuberosity, volar radial ridge, fibrous transition zone, watershed line, and the lunate facet buttress^18-20^. The distal dorsal cortical distance (DDD) and lunate facet distance (LFD) are stricter intra-operative measurements that can be taken with fixed points of measurement. The amount of settling can be predicted regarding the articular surface and volar tilt by monitoring these measurements. It can help surgeons to prevent late altered wrist kinematics^21-23^.

We intended to investigate the relationship between the distal radius collapse and the number of screws used at the metaphyseal part of the plate with the measurement of DDD and LFD in patients treated with a volar locking plate. We hypothesised that loss of reduction after plating is related to the number of distal locking screws when the DDD distance is kept to less than 6mm.

## Materials and Methods

Between July 2015 and June 2019, patients with extra-articular distal radius fractures who had undergone volar locking plating were retrospectively evaluated. This study included 60 patients with distal radius fractures (33 males, 27 females; mean age, 43.5±12.7 years [range, 19-60 years]) who had completed a one-year follow-up. The follow-up period was at least 12 months (range, 12-45 months). According to Osteosynthesefragen/Orthopaedic Trauma Association [AO/OTA] classification, only AO type 23-A2 and 23-A3 fractures that had undergone surgery were analysed in the current study. At least three out of five criteria were required for surgical decision-making: dorsal angulation >20°, shortening >5mm, >50% dorsal comminution, extra-articular fracture, and completion of a rehabilitation program after either operational or conservative care^24^. Patients having intra-articular fractures, operative management other than volar locking plates, open fracture, concomitant same-side upper limb injury, and fractures accompanied by neurovascular deficits were excluded from the study. The research was designed retrospectively following the Declaration of Helsinki. This study received Institutional Review Board approval (IRB: 2019-281. 20/9), and patients provided oral and written informed consent.

In this study, we assessed (1) bone union rate (presence of three out of four instances of cortical bridging on AP and lateral radiographs), (2) distal dorsal cortical distance (measured in mm from the tip of the most distal screw to the dorsal rim of the distal radius, taken intra-operatively on a 20° inclined lateral view)), (3) distal dorsal cortical distance (measured in mm), (3) lunate facet distance (LFD), and (4) the number of metaphyseal screws (number of screws zinserted at the distal part of the plate)^20,22,23,25^. Patients were divided into two groups based on the number of screws inserted at the metaphyseal part of the plate. The radial collapse was defined as having more than a ten per cent loss of radial length compared to the previous measurement.

All fractures were closed injuries and none of the patients had expressed osteoporosis. The volar surface of the distal radius was accessed using a trans-FCR approach^26^. The pronator quadratus was raised subperiosteally along its radial border and retracted, while the thumb and finger flexor tendons were retracted to the lateral side. Under direct visualisation, the fracture was reduced and temporarily fixed with k-wires^20^. Volar plates [CM-Smart; 3.5mm, anatomical volar locking plate. Hipokrat Tibbi, Izmir, Turkey] were applied following fracture reduction. All the inserted distal locking screws were variable-angle screws. In the diaphyseal part, a cortical screw was used in the middle hole of the plate. After plating, a final evaluation was performed using an image intensifier to check that the reduction, plate and screw alignment, and screw length were all good. The wound was closed and a below-elbow slab was applied for two weeks, after which a removable splint was applied and range-of-motion exercises began.

**Fig. 1.**
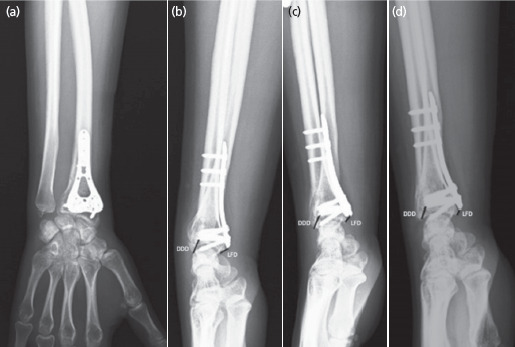
Method of measuring the radiographic parameters of the distal radius fractures: Radial collapse was observed in the radiographs taken during the one-year follow-up period. (a, b) Antero-posterior and lateral view at the time of discharge. (c) Lateral radiograph after three months. (d) Lateral radiograph at one year follow-up.

Standard AP and lateral radiographs of the injured wrist were analysed at the time of initial evaluation, post-operatively, and at follow-ups on the 1st day and at 3rd, 6th and 12th months. Images were assessed using digital radiology software [Agfa HealthCare Impax 6, Belgium]. The first and second authors validated all measurements. If there was more than a ten per cent difference between measurements, the radiologist repeated the measurement. The interobserver kappa value of 0.65 showed good reproducibility. On lateral radiographs, distal dorsal cortex distance and lunate facet distance were measured (Fig. 1). The number of screws used in the metaphyseal region was assessed.

Statistical analysis was performed using the IBM SPSS Statistics for Windows, Version 18. The conformity of continuous variables to normal distribution was evaluated using visual (histogram and probability graphs) and analytical methods (Kolmogorov- Smirnov / Shapiro-Wilk tests). The categorical variables included in the study are presented with frequency, percentage, and continuous variables and mean, standard deviation, maximal and minimal values. Mann Whitney u test was used for comparison of data sets that were not normally distributed for the variables. The statistical significance threshold was accepted as 0.05. Sample size calculation: Cohen's f2 effect size was 0.685, implying that a minimum of 54 fractures would be needed to achieve a power of more than 80% and a threshold of significance of 0.05. So, 60 patients were included in this study.

## Results

The investigated group consisted of 60 patients (27 (45%) female and 33 (55%) male). The mean age was 43.5±12.7 years (range 19-60 years). The mean time of hospitalisation was 4.5 days. The minimum follow-up of patients was 12 months (range 12-45 months). Demographic data are displayed in Table I.

**Table I:** Descriptive characteristics of patients with distal radius fractures undergone volar locking plate fixation

Variable	Mean (SD) / n (%)
Patient	
Mean age (SD)	43.18±12
Female / Male	27 (45%) / 33 (55%)
Injury	
High energy mechanism	12 (20%)
Right wrist fractured	35 (58.3%)
AO fracture classification	
A2	24 (40%)
A3	36 (60%)
The number of distal screws	
≤ 6 screws	32 (53.3%)
≥ 7 screws	28 (46.7%)

Delayed union and non-union were not observed. No significant problem was found related to screw penetration through the dorsal radial cortex and related tendon irritation leading to the removal of implants. Intra-articular screw penetration was not observed.

In all patients, both distal rows of the plate were used depending on surgeon preference. The average number of screws used in the distal part of the plate was 6.4 (minimum 4 - maximum 9). There was a statistically significant improvement in the radiographic parameters after anatomical reduction.

The changes in LFD and DDD measurement were compared according to the number of screws. Since the average number of screws used in patients was calculated as 6.4, the grouping was classified as six screws and below and seven screws and above (Table II; Fig. 1). Accordingly, the changes observed in 6-month and 12-month DDD measurements were less in the group with seven or more screws used (p <0.05). Moreover, the changes observed in 12-month LFM measurements in patients using seven or more screws were statistically significant (p<0.05).

**Table II:** Evaluation of the radial collapse based on the number of screws used in the metaphyseal region

Time (month)	LFM (mm)			DDD (mm)		
	<6 metapyseal screw	>7 metaphyseal screw	*P* value*	<6 metapysealscrew	>7 metaphyseal screw	*P* value*
3	0.11±0.17	0.17±0.29	0.881	0.37±0.36	0.17±0.27	0.085
6	0.37±0.27	0.42±0.33	0.393	0.93±0.77	0.58±0.45	0.001
12	0.76±0.39	0.50±0.37	0.045	0.16±0.90	0.74±0.78	0.018

*: Mann-Whitney U test

## Discussion

The goals of treatment in distal radius fractures are anatomical reduction, maintenance of joint stability, restoration of alignment, achievement of fracture union, pain-free range of motion, and absence of any wound complications.

As a result of our study, we documented the advantage of using more than six screws compared to fewer than six for metaphyseal fragment fixation. Second, the most important findings for providing stability in volar plating were keeping DDD less than 6mm during insertion of the distal screws. The clinical relevance of our results is that distal dorsal cortex distance and the number of screws implemented at the distal part of the plate are modifiable risk factors, and loss of reduction depends on surgical accuracy. According to many clinical and biomechanical trials, fixation of the distal part of the fracture with more screws provides optimal metaphyseal support and creates a scaffold to allow two-plane fixation of distal fragments^27,28^.

Orbay and Touhami have emphasised the clinical importance of the watershed line^19^. In our study, we performed the volar plate application based on these recommendations. This was true, especially for the evaluation of the lunate facet buttress, where we used the lunate facet distance. We tried to locate the screws as distal as possible, avoiding violation of the lunate fossa morphometry. We observed that collapses were fewer in these cases in our clinical study.

In the cadaver study, Mehling *et al* stated that three screws in the distal part created an unstable condition and there should be at least four screws in the distal fragment, and two of these screws should be in a different direction (multidirectional screw)^28^. In addition, they stated that the screws were more durable than pegs. They have seen high resistance (stiffness) in those using seven variable angle screws. In our study, the number of screws used in the metaphyseal part was found to be a minimum of four and a maximum of nine screws. According to the number of screws, the lunate facet distance and the DDD decreases were compared. Since the average number of screws used in patients was 6.4, subgroups were defined as “6 screws and below” and “7 screws and above”. Accordingly, there was a statistically significant difference between 6-month and 12-month DDD collapse between the groups (p<0.05). It was observed that volar plates with seven or more screws had less DDD decrease and fewer radial collapses.

Vosbikian *et al* found that patients with a DDD distance of less than 6mm had fewer collapses in early post-operative and one-year post-operative follow-ups^22^. In our study, we measured the DDD distance of the patients on the first postoperative day and at 3, 6, and 12 months. We observed that all patients with a DDD value of less than 6mm postoperatively had fewer collapses at the 6th and 12th months. We measured lunate facet distance on all patients on the first post-operative day and at 3, 6, and 12 months. When the decrease in LFM measurements of these patients was assessed, there was no statistically significant difference in the reduction in LFM value between the groups at 3 and 6 months post-operatively, while there was a significant difference between the groups at 12 months (p<0.05). At the end of the year, it was observed that the LFM value and the radial collapse were less in the group with more than six metaphyseal screws in place.

The appeal of the variable-angle locking plate lies in the increased versatility for fracture fixation offered to the surgeon. It may have advantages even if the plate is positioned improperly or the patient’s anatomy is distorted. The variable-angle plate has superior strength and stiffness and allows anatomic reduction with low complication rates, including low rates of secondary loss of reduction^29,30^. These observations are supported by the results of our study.

Our research has some limitations. The study was conducted based on retrospective data. However, we did have distinct and comparable groups with appropriate follow-up and no missing data thanks to matching and strict inclusion criteria. The results of our study are also supported by other studies^22,23,28,31^.

We did not assess clinical outcomes in our patient population because it was out of the scope of our purpose. Although screw lengths were not provided for statistical comparison, the surgeon used the same screw length selection techniques. A prospective setting and an incentive for patients to be available for follow-up checks would be preferred for future investigations.

## Conclusions

Our study showed that to prevent post-operative loss of reduction in fractures plated with volar locking distal radius plates, the distal screws placed must be more than seven and DDD, LDF distance should be kept as less as possible.

## References

[1] Jupiter JB, Fernandez DL, Toh CL, Fellman T, Ring D (1996). Operative treatment of volar intra-articular fractures of the distal end of the radius. J Bone Joint Surg Am..

[2] Chen NC, Jupiter JB (2007). Management of distal radial fractures. J Bone Joint Surg Am..

[3] Mulders MAM, van Eerten PV, Goslings JC, Schep NWL (2017). Non-operative treatment of displaced distal radius fractures leads to acceptable functional outcomes, however at the expense of 40% subsequent surgeries. Orthop Traumatol Surg Res..

[4] Stoffel K, Dieter U, Stachowiak G, Gachter A, Kuster MS (2003). Biomechanical testing of the LCP--how can stability in locked internal fixators be controlled?. Injury..

[5] Sommer C, Babst R, Muller M, Hanson B (2004). Locking compression plate loosening and plate breakage: a report of four cases. J Orthop Trauma..

[6] Kandemir U, Augat P, Konowalczyk S, Wipf F, von Oldenburg G, Schmidt U (2017). Implant Material, Type of Fixation at the Shaft, and Position of Plate Modify Biomechanics of Distal Femur Plate Osteosynthesis. J Orthop Trauma..

[7] Heyland M, Duda GN, Mardian S, Schutz M, Windolf M (2017). Stahl oder Titan bei der Osteosynthese : Eine mechanobiologische Perspektive [Steel or titanium for osteosynthesis : A mechanobiological perspective]. Unfallchirurg..

[8] Tank JC, Schneider PS, Davis E, Galpin M, Prasarn ML, Choo AM (2016). Early Mechanical Failures of the Synthes Variable Angle Locking Distal Femur Plate. J Orthop Trauma..

[9] Gautier E, Sommer C (2003). Guidelines for the clinical application of the LCP. Injury..

[10] Potter BK (2016). From Bench to Bedside: How Stiff is Too Stiff? Far-cortical Locking or Dynamic Locked Plating May Obviate the Question. Clin Orthop Relat Res..

[11] Kaab MJ, Frenk A, Schmeling A, Schaser K, Schutz M, Haas NP (2004). Locked internal fixator: sensitivity of screw/plate stability to the correct insertion angle of the screw. J Orthop Trauma..

[12] Wagner M (2003). General principles for the clinical use of the LCP. Injury..

[13] Smith WR, Ziran BH, Anglen JO, Stahel PF (2007). Locking plates: tips and tricks. J Bone Joint Surg Am..

[14] Mattila VM, Huttunen TT, Sillanpaa P, Niemi S, Pihlajamaki H, Kannus P (2011). Significant change in the surgical treatment of distal radius fractures: a nationwide study between 1998 and 2008 in Finland. J Trauma..

[15] Mellstrand-Navarro C, Pettersson HJ, Tornqvist H, Ponzer S (2014). The operative treatment of fractures of the distal radius is increasing: results from a nationwide Swedish study. Bone Joint J..

[16] Talmaç MA, Gorgel MA, Kanar M, Tok O, Ozdemir HM (2019). Comparison of three surgical methods in the treatment of intraarticular comminuted distal radius fractures: Volar locking plate, non-bridging external fixator, and bridging external fixator. Eklem Hastalik Cerrahisi..

[17] Aldemir C, Onder M, Dogan A, Duygun F, Oguz N (2015). Lunat fossanın morfometrik anatomik calismasi ve klinik onemi [Morphometric anatomic study and clinical significance of lunate fossa]. Eklem Hastalik Cerrahisi..

[18] Imatani J, Akita K, Yamaguchi K, Shimizu H, Kondou H, Ozaki T (2012). An anatomical study of the watershed line on the volar, distal aspect of the radius: implications for plate placement and avoidance of tendon ruptures. J Hand Surg Am..

[19] Orbay JL, Touhami A (2006). Current concepts in volar fixed-angle fixation of unstable distal radius fractures. Clin Orthop Relat Res..

[20] Aldemir C, Duygun F (2017). Is it possible to avoid intra-articular screw penetration with minimal use of fluoroscopy in the application of distal radius volar plate?. Eklem Hastalik Cerrahisi..

[21] Linscheid RL (1986). Kinematic considerations of the wrist. Clin Orthop Relat Res..

[22] Vosbikian MM, Ketonis C, Huang R, Ilyas AM (2016). Optimal Positioning for Volar Plate Fixation of a Distal Radius Fracture: Determining the Distal Dorsal Cortical Distance. Orthop Clin North Am..

[23] Andermahr J, Lozano-Calderon S, Trafton T, Crisco JJ, Ring D (2006). The volar extension of the lunate facet of the distal radius: a quantitative anatomic study. J Hand Surg Am..

[24] Boszotta H, Helperstorfer W, Sauer G (1991). Zur Operationsindikation bei der distalen Radiusfraktur [Indications for surgery in distal radius fractures]. Unfallchirurg..

[25] Lichtman DM, Bindra RR, Boyer MI, Putnam MD, Ring D, Slutsky DJ (2011). American Academy of Orthopaedic Surgeons clinical practice guideline on: the treatment of distal radius fractures. J Bone Joint Surg Am..

[26] Ring D, Jupiter JB (1999). Operative exposure of fractures of the distal radius. Tech Hand Up Extrem Surg..

[27] Inagaki K, Kawasaki K (2016). Distal radius fractures-Design of locking mechanism in plate system and recent surgical procedures. J Orthop Sci..

[28] Mehling I, Muller LP, Delinsky K, Mehler D, Burkhart KJ, Rommens PM (2010). Number and locations of screw fixation for volar fixed-angle plating of distal radius fractures: biomechanical study. J Hand Surg Am..

[29] Mehling I, Meier M, Schlor U, Krimmer H (2007). Multidirektionale winkelstabile Versorgung der instabilen distalen Radiusfraktur [Multidirectional palmar fixed-angle plate fixation for unstable distal radius fracture]. Handchir Mikrochir Plast Chir..

[30] Vlcek M, Visna P (2008). Sestimesicni funkcni a rentgenove vysledky zlomenin distalniho radia osetrenych multidirekcionalnimi zamykatelnymi dlahami [Six-month functional and X-ray outcomes of distal radius fractures managed using multidirectional locking plates]. Rozhl Chir..

[31] Drobetz H, Black A, Davies J, Buttner P, Heal C (2018). Screw placement is everything: Risk factors for loss of reduction with volar locking distal radius plates. World J Orthop..

